# Nanochitin/MXene Composite Coated on Quartz Crystal Microbalance for Humidity Sensing

**DOI:** 10.3390/nano13243135

**Published:** 2023-12-14

**Authors:** Yanqi Li, Xianhe Huang, Qiao Chen, Yao Yao, Wei Pan

**Affiliations:** 1 School of Automation Engineering, University of Electronic Science and Technology of China, Chengdu 611731, China; liyanqi@std.uestc.edu.cn; 2 School of Automation, Chongqing University of Posts and Telecommunications, Chongqing 400065, China; 3 College of Communication Engineering, Chengdu University of Information Technology, Chengdu 610225, China; yyao@cuit.edu.cn; 4 Zhejiang Academy of Special Equipment Science, Hangzhou 310005, China

**Keywords:** humidity sensor, quartz crystal microbalance (QCM), nanochitin, Ti_3_C_2_T_x_ MXene, high quality factor

## Abstract

MXenes, as a typical graphene-like material, excels in the realm of humidity sensing owing to its two-dimensional layer structure, high electrical conductivity, tunable chemical properties, hydrophilicity, and large specific surface area. This study proposed a quartz crystal microbalance (QCM) humidity sensor using a nanochitin/Ti_3_C_2_T_x_ MXene composite as a humidity-sensing material. The morphology, nanostructure, and elemental composition of nanochitin, Ti_3_C_2_T_x_ MXene, and nanochitin/Ti_3_C_2_T_x_ MXene composite materials were characterized using transmission electron microscopy, Fourier transform infrared spectroscopy, and X-ray diffraction. Compared to the pure Ti_3_C_2_T_x_ MXene-coated QCM humidity sensor, the nanochitin/Ti_3_C_2_T_x_ MXene-coated QCM humidity sensor exhibited a higher sensitivity (20.54 Hz/%RH) in the humidity range of 11.3% to 97.3%. The nanochitin/Ti_3_C_2_T_x_ Mxene-coated QCM humidity sensor also demonstrated low humidity hysteresis (2.12%RH), very fast response/recovery times (4.4/4.1 s), a high quality factor (37 k), and excellent repeatability and sustained stability over time. Eventually, a bimodal exponential kinetics adsorption model was utilized for the analysis of the response mechanism of the nanochitin/Ti_3_C_2_T_x_ MXene composite material-based QCM humidity sensor. This study provides new ideas for optimizing the moisture-sensitive performance of MXene-based QCM humidity sensors.

## 1. Introduction

Accurate humidity measurement holds significant importance in various fields, such as agricultural production and storage, industrial manufacturing, food processing, healthcare, and meteorology [1,2,3,4]. With the advancement of technology, researchers have employed various methods to enhance the performance parameters of humidity sensors, including resistors [5,6], capacitors [7,8], field-effect transistors (FET) [9,10], and quartz crystal microbalances (QCMs) [11,12]. Among these, the QCM transducer has gained popularity as a favored candidate in the realm of humidity sensing owing to its high sensitivity to nanoscale changes in water molecule mass, strong interference rejection capability, excellent stability, and digital output [13,14,15,16]. The QCM sensor is based on the classical Sauerbrey equation [17]:

(1) $$\Delta f=-\frac{2f_{0}^{2}}{A\sqrt{\rho_{q}\mu_{q}}}\Delta m$$

In the equation, $f_{0}$ represents the fundamental resonance frequency of the quartz crystal, $\rho_{q}$ and $\mu_{q}$ are the density and shear modulus of the quartz crystal, respectively, and A denotes the electrode area. The mass variation of the surface load on the quartz crystal (Δm) is equivalently converted into a frequency change (Δf) of the quartz crystal’s resonance. However, the electrode can only adsorb a small number of water molecules, resulting in a low-frequency shift of the QCM. To address this issue, researchers have proposed adding a specific sensing material on the surface of the QCM crystal to adsorb water molecules. Different materials exhibit varying degrees of adsorption strength towards water molecules. The sensitivity of humidity sensors can be enhanced by altering the electrode coating material, thereby modifying the mass of water molecules adsorbed on the QCM’s surface and enabling the fabrication of humidity sensors with higher sensitivity. In the past few years, a rising number of researchers have been exploring hydrophilic materials [18] or incorporating other micro-particles into humidity-sensitive materials at the microstructural level to enhance the adsorption capacity of the materials for water molecules [12,16,19,20].

In recent years, there has been increasing interest in utilizing two-dimensional materials with a high specific surface area and intricate surface chemistry as highly promising sensor materials among the diverse range of materials studied in the field [21,22]. Among all two-dimensional materials, transition metal carbides/nitride (MXene) has emerged as a prominent candidate. Ti_3_C_2_T_x_ is the first Mxene that was synthesized by the Gogotsi group through room-temperature etching of Ti_3_AlC_2_ in HF [23,24]. T represents functional groups including =O, -OH, -F, etc., and x represents the quantity of these groups. MXene exhibits higher mechanical strength, higher specific surface area, and better chemical stability compared to graphene [25,26,27]. Following HF acid etching, MXene possesses a chemically active surface with abundant hydroxyl (-OH) and fluorine (-F) groups, rendering it a highly hydrophilic surface. Moreover, its highly tunable surface functional groups facilitate its compatibility with other materials, making MXene-based sensitive films hold great potential in the field of humidity-sensitive materials [28]. Shpigel et al. [29] investigated the water adsorption characteristics of MXene electrodes when immersed in various electrolyte solutions. An et al. [25] examined the mechanism underlying the humidity response of MXene/polyelectrolyte multilayer films. This response is attributed to the modulation of film thickness and interlayer spacing by water molecules, consequently influencing the tunneling resistance between MXene sheets. Li et al. [30] developed a QCM humidity sensor based on MXene, which exhibited a sensitivity of 12.8 Hz/%RH, response/recovery times of 6 s/2 s, and a hysteresis of only 1.16%RH. Li et al. [14] fabricated QCM humidity sensors using Ti_3_C_2_T_x_ MXene, alkali-treated Ti_3_C_2_T_x_, and sulfurized Ti_3_C_2_T_x_ via spin coating. Among these sensors, the QCM humidity sensor based on sulfurized Ti_3_C_2_T_x_ demonstrated exceptional sensitivity of 105 Hz/%RH at ambient temperature, accompanied by rapid response and recovery times of 13 s and 6 s, respectively.

However, as a two-dimensional nanosheet with a high aspect ratio and large specific surface area, MXene possesses inherent structural characteristics that enable it to self-stack. Furthermore, the loss of surface functional groups during synthesis or environmental exposure diminishes electrostatic repulsion, promoting stacking behavior. Coupled with the strong van der Waals forces, self-stacking phenomena are prone to occur [31,32,33], which limits the contact between internal active sites and water molecules. Chitin, derived from crab and shrimp shells, is a natural polysaccharide and the second most abundant biopolymer on Earth [34]. Nanochitin, as a nanomaterial, offers unique characteristics due to its nano-sized structure derived from chitin. Understanding its physiochemical properties is crucial as nanochitin exhibits remarkable properties such as high surface area, biocompatibility, and mechanical strength. Nanochitin has excellent hydrophilic properties due to the presence of a large number of hydroxyl (-OH) and amino (-NH2) hydrophilic groups on its macromolecular chain [35,36,37]. In this study, nanochitin was introduced as an interlayer support material in MXene. The excellent hydrophilicity and dispersibility of nanochitin can help MXene to be better dispersed in an aqueous solution; it can be embedded in the layers of MXene, and nanochitin is usually positively charged, which increases the repulsive force between the layers of MXene to prevent self-stacking. Additionally, the hydrophilic nature of nanochitin provides more active sites for the humidity-sensitive film [38]. The nanochitin/Ti_3_C_2_T_x_ MXene composite material was first prepared, and then, the deposition of the humidity-sensitive material onto the central region of the QCM electrode was achieved through the drop-casting method. The performance parameters of the QCM humidity sensor, including sensitivity, humidity hysteresis, response/recovery time, repeatability, and stability, were evaluated using a saturated salt solution humidity chamber. Ultimately, an in-depth analysis was conducted to elucidate the humidity-sensing mechanism of the nanochitin/Ti_3_C_2_T_x_ MXene coating employed on the QCM humidity sensor.

## 2. Materials and Methods

### 2.1. Fabrication of QCM Humidity Sensors

**Figure 1:**
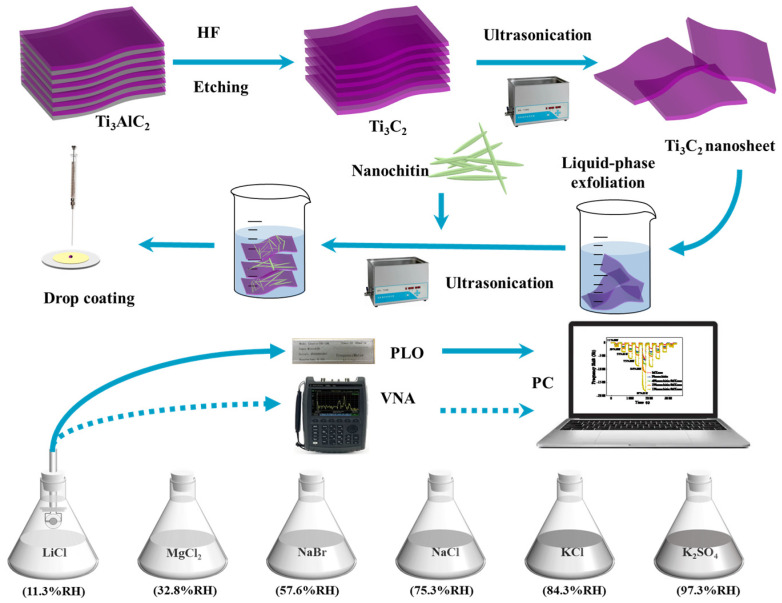
Schematic diagram of the nanochitin/Ti_3_C_2_T_x_ MXene humidity sensor preparation process and detection device.

The QCM was repeatedly cleaned with deionized water and acetone, followed by drying in a drying oven at 60 °C for future use. The preparation process of the nanochitin/Ti_3_C_2_T_x_ MXene composite material is depicted in Figure 1. Bulk Ti_3_AlC_2_ MAX (4.0 g) was slowly added to a 40 wt% HF etching solution (80 mL) and stirred magnetically at ambient temperature for 24 h. At 15 °C, ultrasonic oscillation was applied using a constant temperature ultrasonic device for 3 h. The Ti_3_C_2_T_x_ MXene nanosheets were collected via centrifugation at 10,000 rpm for 30 min. A Ti_3_C_2_T_x_ MXene solution with a concentration of 1 mg/mL was prepared, and 5 mg, 10 mg, and 20 mg of nanochitin powder were added to a separate 10 mL of the Ti_3_C_2_T_x_ MXene composite solution. The solution was sonicated for 2 h at 15 °C to obtain a homogeneous solution, which was named NCM-A, NCM-B, and NCM-C, respectively. Following this, the sensitive material was meticulously deposited at the central region of the QCM electrode utilizing a facile drop-coating technique until the sensitive material dried and adhered to the electrodes, thereby enabling the fabrication of the QCM humidity sensor.

### 2.2. Devices

Figure 1 illustrates the experimental configuration, comprising a quartz crystal microbalance (QCM), vector network analyzer (VNA), phase-locked oscillator (PLO), humidity bottles, and personal computer (PC). The AT-cut QCM with a fundamental frequency of 6 MHz was obtained from Wuhan Haichuang Electronics Co., Ltd., Wuhan, China, with the diameters of quartz crystal and silver electrodes are 8 mm and 5 mm, respectively. The PLO and VNA used in the experiment were acquired from Leopboard Technologies Co., Ltd. (Chengdu, China). The phase-locked oscillator serves as an excitation circuit designed to measure the resonant frequency of the nanochitin/Ti_3_C_2_T_x_ MXene composite humidity sensor, facilitating the real-time acquisition of the dynamic frequency changes of the QCM sensor. Meanwhile, the vector network analyzer was utilized to analyze the resonant behavior and conductance spectrum of the QCM humidity sensor. Subsequently, the frequency signals and resonance characteristics were transferred to the personal computer (PC) for meticulous data acquisition and in-depth analysis. Different relative humidity levels were generated using saturated solutions of K_2_SO_4_, KCl, NaCl, NaBr, MgCl_2_, and LiCl at 25 °C, corresponding to 97.3%, 84.3%, 75.3%, 57.6%, 32.8%, and 11.3% relative humidity, individually. All experiments were conducted at ambient temperature (25 °C). Bulk Ti_3_AlC_2_ MAX, nanochitin, hydrofluoric acid (HF, 40 wt%), potassium sulfate (K_2_SO_4_), potassium chloride (KCl), sodium chloride (NaCl), sodium bromide (NaBr), magnesium chloride (MgCl_2_), and lithium chloride (LiCl), were purchased from Shanghai Aladdin Biochemical Polytron Technologies Inc. (Shanghai, China). All the solvents and chemicals were of analytical grade and used without further purification).

## 3. Results and Discussion

### 3.1. Structural and Morphological Features

**Figure 2:**
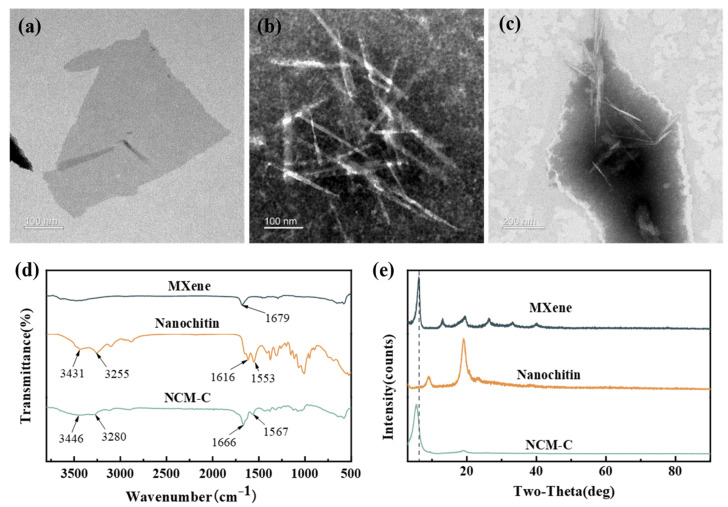
TEM images of (**a**) Ti_3_C_2_T_x_ MXene, (**b**) nanochitin, and (**c**) NCM-C composite. (**d**) FTIR spectra and (**e**) XRD image of Ti_3_C_2_T_x_ MXene, nanochitin, and NCM-C.

Figure 2a–c exhibit the observed images of Ti_3_C_2_T_x_ MXene, nanochitin, and NCM-C composite materials using transmission electron microscopy (CM10, Philips, Eindhoven, The Netherlands). Figure 2a reveals the two-dimensional sheet-like structure of Ti_3_C_2_T_x_ MXene. Figure 2b demonstrates that nanochitin consists of needle-shaped microcrystals with lengths ranging from tens to hundreds of nanometers. Incorporating nanochitin into Ti3C2Tx MXene effectively prevents the self-stacking of Ti_3_C_2_T_x_ MXene, as shown in Figure 2c. Figure 2d characterizes the surface functional groups of Ti_3_C_2_T_x_ MXene, nanochitin, and NCM-C through Fourier transform infrared spectroscopy (Nicolet iS10, Thermo Fisher Scientific Inc., Waltham, MA, USA). Nanochitin exhibits two characteristic peaks at 1616 cm^−1^ and 1553 cm^−1^, mainly attributed to the stretching of the C=O bond in amide I and the combined vibrations of C-N and N-H in amide bending [39]. The presence of abundant hydrophilic groups in nanochitin is indicated by the two distinct peaks observed at 3431 cm^−1^ and 3255 cm^−1^, corresponding to the stretching vibrations of O-H and N-H, respectively [39,40]. Furthermore, analysis of the FTIR spectra of NCM-C indicates the presence of characteristic peaks of both nanochitin and MXene, confirming the successful preparation of the composite material. In Figure 2e, through X-ray diffraction (D8 ADVANCE, Bruker, Bremen, Germany), a prominent peak is observed at 6.19° for Ti_3_C_2_T_x_ MXene, corresponding to the (002) crystal plane of Ti_3_C_2_T_x_ MXene [41], without significant impurity peaks, indicating an ideal etching effect and the desired Ti_3_C_2_T_x_ MXene solution. The presence of a strong peak at 5.57° for NCM-C indicates the leftward shift of the main peak of the (002) crystal plane and increased interlayer spacing in the composite film [42].

### 3.2. Sensitivity

**Figure 3:**
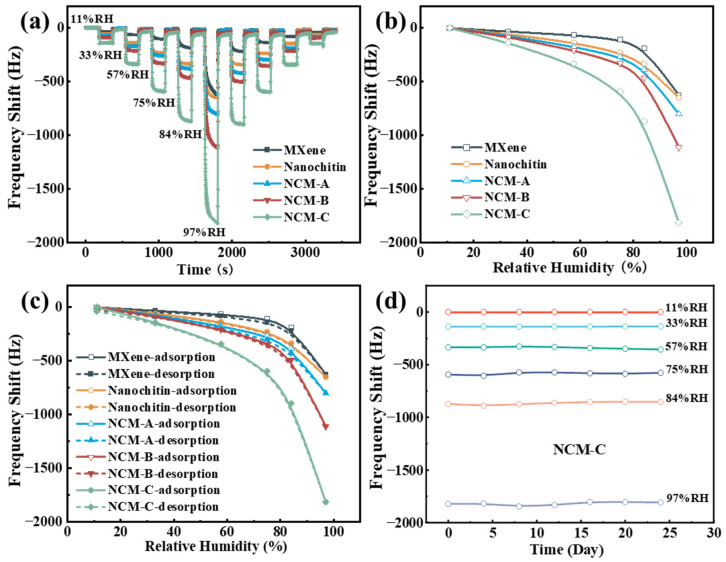
(**a**) Dynamic characteristics, (**b**) sensitivity, and (**c**) humidity hysteresis. (**d**) Long-term stability of NCM-C.

In the domain of humidity sensors, sensitivity refers to the magnitude of change in the sensor’s output signal corresponding to variations in humidity. It is typically quantified as the ratio of the change in the sensor’s output signal (frequency shift Δf) to the relative humidity level ΔRH. Figure 3a illustrates the dynamic frequency response of the five QCM sensors prepared in this experiment under different humidity conditions. As shown in Figure 3b, within the humidity range of 11.3% to 97.3%, the maximum frequency shifts of Ti_3_C_2_T_x_ MXene, nanochitin, NCM-A, NCM-B, and NCM-C are measured as 624.89 Hz, 632.51 Hz, 785.9 Hz, 1097 Hz, and 1766.61 Hz, respectively. The corresponding sensitivities are calculated as 7.27 Hz/%RH, 7.35 Hz/%RH, 9.14 Hz/%RH, 12.76 Hz/%RH, and 20.54 Hz/%RH. These results demonstrate a substantial improvement in the sensitivity of the QCM humidity sensor based on Ti_3_C_2_T_x_ MXene upon incorporating nanochitin.

### 3.3. Humidity Hysteresis and Stability

Humidity hysteresis refers to the disparity or delay in the output signal of a humidity sensor during the process of increasing and decreasing humidity levels. This phenomenon typically arises from discrepancies in the humidity adsorption and desorption processes of the sensor’s materials. The presence of humidity hysteresis can lead to inaccuracies and instability in humidity measurements. Consequently, minimizing hysteresis is a crucial objective in the design and optimization of humidity sensors. As shown in Figure 3c, within the range of 11.3% RH to 97.3% RH, the maximum frequency differences for Ti_3_C_2_T_x_ MXene, nanochitin, NCM-A, NCM-B, and NCM-C are measured as 33.56 Hz, 13.16 Hz, 39.31 Hz, 28.29 Hz, and 38.6 Hz, respectively. The corresponding humidity hysteresis values are calculated as 5.37% RH, 2.02% RH, 4.90% RH, 2.54% RH, and 2.12% RH. The Ti_3_C_2_T_x_ MXene-based QCM humidity sensor exhibits a significantly higher humidity hysteresis compared to the nanochitin-based humidity sensor. The introduction of nanochitin into Ti_3_C_2_T_x_ MXene enhances the sensitivity and concurrently reduces the humidity hysteresis of the humidity sensor.

As can be seen from the experimental results, all five groups show excellent repeatability. Frequency response tests were performed at various humidity levels every 4 days for a duration of 24 days to assess the long-term stability of the NCM-C sensor. The results demonstrate that there is no significant frequency change, indicating excellent long-term stability, as shown in Figure 3d.

### 3.4. Response/Recovery Times

**Figure 4:**
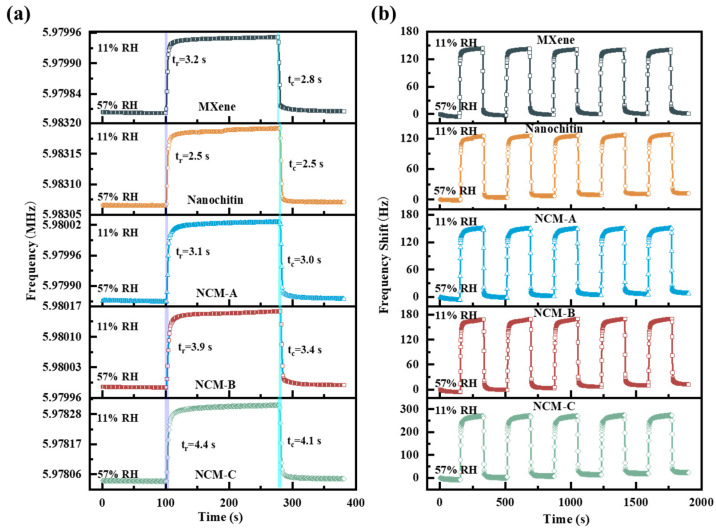
(**a**) The response/recovery times and (**b**) the repeatability times of Ti_3_C_2_T_x_ MXene, nanochitin, NCM-A, NCM-B, and NCM-C from 57.6% RH (ambient humidity) to 11.3% RH.

In the ambient environment, the humidity-sensitive film of the QCM humidity sensor undergoes a continuous process of water absorption and desorption. If the environment is stable, these two processes gradually reach dynamic equilibrium. In practical humidity measurement, changes in the environmental humidity induce alterations in the frequency response of the sensor. Because the frequency change in the QCM sensor is typically exponential, the response/recovery time refers to the time required for the sensor to respond to humidity changes and reach a stable state at 63.2% of the final value. A shorter response/recovery time indicates better sensor performance. We conducted tests on the five sets of humidity sensors we prepared in a low humidity range of 57%RH to 11%RH. The results shown in Figure 4a indicate that the response times (t_r_) for Ti_3_C_2_T_x_ MXene, nanochitin, NCM-A, NCM-B, and NCM-C are 3.2 s, 2.5 s, 3.1 s, 3.9 s, and 4.4 s, respectively, while the recovery times (t_c_) are 2.8 s, 2.5 s, 3.0 s, 3.4 s, and 4.1 s, respectively. The experimental findings demonstrate a positive correlation between the amount of nanochitin adhered to the surface of Ti_3_C_2_T_x_ MXene and the corresponding increase in sensor sensitivity. However, at the same time, the response/recovery time also becomes longer.

The performance indicators of humidity sensors, such as sensing range, response recovery time, and humidity hysteresis, are common parameters for all humidity sensors. Therefore, in Table 1, we compared the performance indicators of our sensor NCM-C with those of sensors based on other sensing principles reported in the past two years. Table 1 shows that, compared to other published humidity sensors, NCM-C demonstrates excellent low humidity hysteresis performance, as well as relatively good response and recovery times.

### 3.5. Repeatability and Quality Factor

In this experiment, the QCM humidity sensor was subjected to five repeated experiments in humidity bottles with a humidity level of 11%RH, starting from an ambient environment of 57%RH. The frequency variations of all sensors are recorded and graphically illustrated in Figure 4b.

In humidity sensors, the quality factor represents the quality factor of the crystal, which reflects the energy loss of the crystal. The quality factor is closely related to the stability of the QCM humidity sensor. Vig and Walls, among others, have found that a decrease in the quality factor can lead to significant frequency noise [49]. The minimum achievable resolution and frequency noise of a quartz crystal resonator are closely related to the quality factor [50,51]:

(2) $$\frac{\Delta m}{A}=-\frac{\sqrt{\rho_{q}\mu_{q}}}{2f_{0}^{2}}\cdot \frac{9.6\times 10^{-8}f_{0}}{Q}$$

(3) $$\sigma_{y}{\left(\tau \right)}_{min}=\frac{1.0\times 10^{-7}}{Q}$$

As shown in Equations (2) and (3), it can be observed that as the quality factor increases, the frequency noise $\sigma_{y}$ of the quartz crystal resonator decreases, and the minimum achievable resolution becomes higher. This indicates that the resonator becomes more stable.

**Figure 5:**
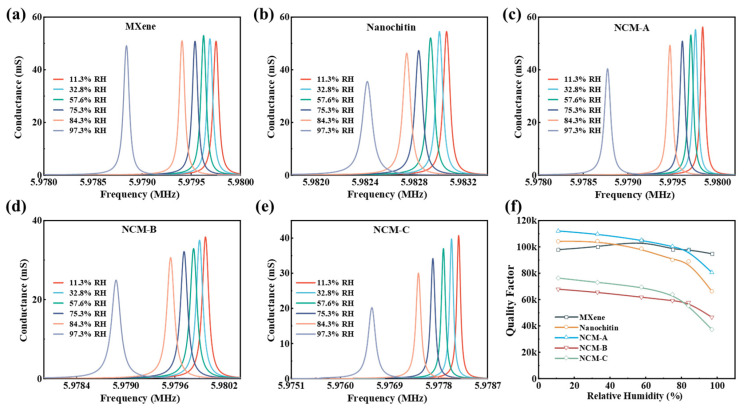
The conductance of (**a**) Ti_3_C_2_T_x_ MXene, (**b**) nanochitin, (**c**) NCM-A, (**d**) NCM-B, and (**e**) NCM-C. (**f**) Quality factors.

Conductance spectrum analysis serves as an effective approach for investigating the electrical characteristics and mechanical properties of QCM sensors. Figure 5a–e present the conductance spectra of Ti_3_C_2_T_x_ MXene and nanochitin-coated QCM sensors measured using a vector network analyzer at different relative humidity levels. When the resonant state is stable, all parameters of the QCM vibration can be detected. As the humidity level rises, the conductance peak of the QCM sensor undergoes a frequency shift towards lower frequencies, accompanied by a gradual widening of the half-bandwidth (HBW). The quality factor (Q) can be defined as the ratio between the maximum frequency value (f) and the HBW:

(4) $$Q=\frac{f}{HBW}$$

Figure 5f presents the quality factor (Q) of the prepared sensors at different environmental humidity levels. The results demonstrate that the quality factor of Ti_3_C_2_T_x_ MXene remains around 100 K without significant changes as the humidity increases. However, with an increased amount of nanochitin, the quality factor decreases more significantly, with nanochitin showing the largest decrease, with a magnitude of 30 k. This phenomenon can be attributed to the enhanced viscoelastic properties of the sensitive film upon water absorption. The higher the amount of nanochitin, the greater the adsorption of water molecules on the film surface, resulting in a larger frequency shift of the sensor. In particular, we observed that the nanochitin in Figure 5b–e, namely NCM-A, NCM-B, and NCM-C, exhibits a significant decrease in frequency when the relative humidity (RH) increases from 84.3% to 97.3%. This phenomenon can be attributed to the rigid nature of the sensitive membrane in low-humidity environments. However, as the humidity rises from 84% RH to 97% RH, the viscosity of the sensitive membrane increases sharply due to water absorption, causing it to lose its rigidity and resulting in a drastic decrease in its quality factor. Nanochitin-coated sensors degrade more than Ti_3_C_2_T_x_ MXene-coated QCM sensors because the mechanical strength (about 300 GPa) of Ti_3_C_2_T_x_ MXene film is superior to the original nanochitin film, resulting in less energy loss. Table 2 compares the humidity-sensitive performance of NCM-C with other QCM humidity sensors reported in the literature. It can be observed that NCM-C exhibits an excellent quality factor and favorable response/recovery time while maintaining a decent sensitivity.

### 3.6. Humidity-Sensing Mechanism

**Figure 6:**
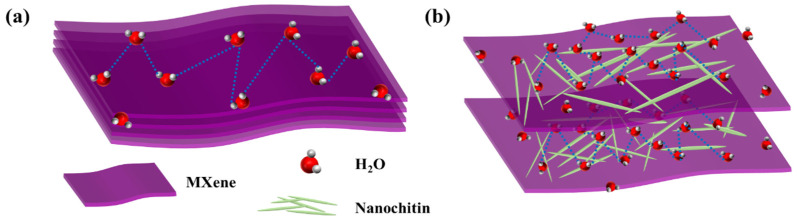
Illustration of the possible humidity-sensing mechanism of (**a**) Ti_3_C_2_T_x_ Mxene and (**b**) nanochitin/Ti_3_C_2_T_x_ MXene.

According to the existing literature discussions, Ti3C2Tx MXene and nanochitin surfaces possess abundant hydroxyl groups, facilitating water molecules’ spontaneous adsorption or desorption [28,39,40]. As depicted in Figure 6, at low humidity levels, a minimal quantity of H_2_O chemically adsorbs onto Ti_3_C_2_T_x_ MXene and nanochitin surfaces, forming a monolayer chemisorption of H_2_O. With an increase in humidity levels, a significant number of water molecules undergo hydrogen bonding between the hydrogen atoms and electronegative oxygen atoms, resulting in their physical adhesion to the material surface. During this process, the variation in the quantity of adhered water molecules leads to changes in the mass of the QCM surface composite material, subsequently influencing the resonant frequency of the QCM.

Ti_3_C_2_T_x_ MXene is prone to self-stacking, making it difficult for water molecules to penetrate. This implies that most active sites of Ti_3_C_2_T_x_ MXene are not exposed, resulting in lower sensitivity. By incorporating nanochitin into Ti_3_C_2_T_x_ MXene, nanochitin adheres to the surface of the monolayer Ti_3_C_2_T_x_ MXene sheets, preventing self-stacking. Moreover, it offers an increased number of active sites, thereby facilitating the adsorption of a larger quantity of water molecules and engendering heightened sensitivity.

The bimodal exponential kinetics adsorption model is a mathematical model used to describe the variation of adsorption rate with time in an adsorption system. In contrast to the common single exponential kinetic model, the bimodal exponential model assumes that the adsorption rate is represented by a linear combination of two exponential terms. This implies that the variation of adsorption rate with time is not a simple exponential decay or increase but is influenced by two distinct exponential terms. The bimodal exponential kinetics adsorption model is typically employed to describe the adsorption kinetics behavior in complex adsorption systems, such as the adsorption process on porous materials or heterogeneous surfaces. As the variation of adsorption rate in these systems may be influenced by multiple factors, the use of the bimodal exponential model allows for a more accurate capture of the rate variation during the adsorption process. The equations for adsorption and desorption of the bimodal exponential kinetics model are as follows [63]:

(5) $$Y=A_{1}e^{\frac{-t}{\tau_{1ADS}}}+B_{1}e^{\frac{-t}{\tau_{2ADS}}}+Y_{0}$$

(6) $$Y=A_{2}e^{\frac{-t}{\tau_{1DES}}}+B_{2}e^{\frac{-t}{\tau_{2DES}}}+Y_{0}$$

In Equations (5) and (6), Y represents the total mass of H_2_O adsorbed by the humidity-sensitive film on the quartz crystal chip at time t, and $Y_{0}$ is the total mass at a stable state. $A_{i}$ and $B_{i}$ (i = 1, 2) represent the amplitudes of the physical and chemical dynamic processes, respectively.$\tau_{1ADS}$ and $\tau_{2ADS}$ represent the characteristic times for adsorption, while $\tau_{1DES}$ and $\tau_{2DES}$ represent the characteristic times for desorption. We performed fitting on the two processes of the NCM-C humidity sensor between 11% RH and 97% RH, resulting in the following fitted curve and equation.

(7) $$\Delta m=-1714.53e^{-t/5.30}-435.68e^{-t/38.11}+1754.53$$

(8) $$\Delta m=2662.05e^{-t/1.96}+47.78e^{-t/59.13}-1607.47$$

**Figure 7:**
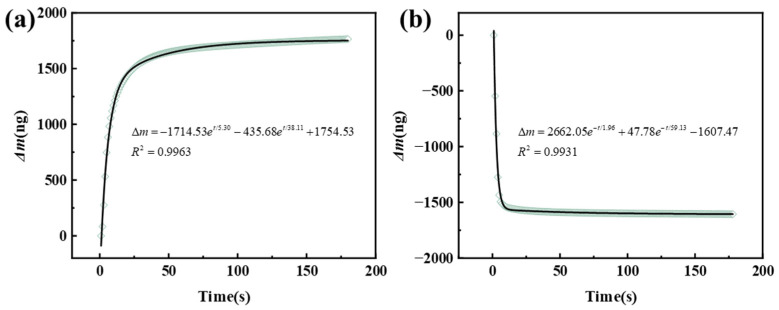
The fitting curve of the modal exponential dynamics model of the NCM-C. (**a**) Adsorption process and (**b**) desorption process.

As shown in Figure 7, the correlation coefficients (R^2^) for the fitted adsorbed and desorbed processes are 0.9963 and 0.9931, respectively. The bimodal exponential kinetics adsorption model effectively fits the data and provides a highly accurate explanation of the adsorption and desorption process of water molecules in the composite membrane. $\tau_{1ADS}$ is 5.30, representing the fast chemical adsorbed process of the first layer of H_2_O, while $\tau_{2ADS}$ is 38.11, typically associated with the slower physical adsorbed process of multilayer water molecules. This observation can be ascribed to the difference in bonding energies between various molecules as well as to the different orientations of water dipoles over longer timescales. In contrast, in the desorption process, $\tau_{1DES}$ and $\tau_{2DES}$ -are 1.96 and 59.13, respectively, which could be assigned to the quick desorbed process of physically adsorbed H_2_O and the slower desorbed process of chemisorbed monolayers [64].

## 4. Conclusions

In summary, this study fabricated a QCM humidity sensor based on a nanochitin/Ti_3_C_2_T_x_ MXene composite material. The introduction of the hydrophilic material nanochitin into Ti_3_C_2_T_x_ MXene effectively prevented self-stacking and improved the sensitivity of the QCM humidity sensor. In comparison, the NCM-C QCM humidity sensor exhibited the highest sensitivity (20.54 Hz/%RH), negligible humidity hysteresis (2.12% RH), rapid response and recovery time (4.4 s/4.1 s), excellent repeatability, and stability. Furthermore, the study combined a bimodal exponential kinetics adsorption model to elucidate the humidity-sensing mechanism of the nanochitin/Ti_3_C_2_T_x_ MXene composite material QCM humidity sensor. In the future, we plan to conduct in-depth research on the surface morphology, the thickness of the active layer, and the hydrophilicity/hygroscopicity of the material, continuing to explore its humidity-sensing mechanism. The designed sensor in this work holds great potential as a promising candidate for future humidity-sensing applications.

## Figures and Tables

**Table 1:** Comparative performance evaluation of humidity sensors based on different sensing principles.

Sensing Principle	Materials	Sensing Range (%RH)	Hysteresis (%RH)	Res./Rec. Time (s)	Ref.
Resistance	Gelatin thin fi	15–86	/	4/6.3	[43]
Impedance	Ni-Co-P	0–97.5	3	95/27	[44]
Resistance	CERP	0–100	/	5/16	[45]
Impedance	Self-supported polymer	11–95	8.5	12.5/>100 s	[46]
Capacitance	Purple sweet potato peel	0–85	5	1/2	[47]
Impedance	PVA/GF	40–90	/	2/3.2	[48]
Frequency shifts	NCM-C	11.3–97.3	2.12	4.4/4.1	This work

**Table 2:** Performance of nanochitin/Ti_3_C_2_T_x_ Mxene-coated QCM humidity sensors compared to other QCM humidity sensors.

Materials	Sensing Range (%RH)	Sensitivity (Hz/%RH)	Hysteresis (%RH)	Res./Rec. Time (s)	Q	Ref.
ND/MWCNT	11.3–97.3	23.50	2	3/2.5	23 k	[52]
S-Ti_3_C_2_	11.3–97.3	12.8	1.16	6/2	/	[30]
NCNCs	11.3–84.3	25.6	5.9	18/10	/	[53]
Graphite	11.3–97.3	2.38	/	8/5	43 k	[54]
PDA@CNC/GO30	11.3–97.3	54.66	4.3	37/5	/	[55]
PANI/GO	0–97.3	20.20	/	13/2	6 k	[56]
BiOCl	11.3–97.3	7.3	2	5.2/4.5	/	[57]
ZnS	22–97	10	/	42/259	/	[58]
Lignin	11.3–97.3	61	6.2	28/5	1.5 k	[59]
PPy/SnS_2_	11.3–97.3	29.0	/	21/4	12 k	[60]
GO	6.4–93.5	22.1	/	45/24	/	[61]
GO/PEI	11.3–97.3	27.25	0.54	53/18	/	[62]
NCM-C	11.3–97.3	20.54	2.12	4.4/4.1	37 k	This work

## Data Availability

Data are contained within the article.
